# Leiomyosarcoma of the chest wall mimicking schwannoma resected by a video-assisted thoracoscopic approach: a case report

**DOI:** 10.1093/jscr/rjab563

**Published:** 2022-01-20

**Authors:** Saki Yamamoto, Masahide Hirose, Takeshi Oyaizu, Aya Muramatsu, Makoto Suzuki, Shinichiro Ohta

## Abstract

Chest wall sarcomas account for <20% of all soft tissue sarcomas of which leiomyosarcomas represent only 1–4%. We report a case of thoracic leiomyosarcoma that resembled schwannoma in preoperative image studies. A 79-year-old man presented to our hospital with a chest wall tumor that increased in size over 3 months. Computed tomography of the chest revealed a 3-cm mass arising from the chest wall. Thoracic magnetic resonance imaging showed a solid tumor that was hypo-intense on T1-weighted imaging and iso-intense on T2-weighted imaging. Chest wall resection was performed using a video-assisted thoracoscopic approach after a frozen section examination revealed sarcoma. The histological diagnosis was leiomyosarcoma. Liver and multiple lung metastases were detected 5 years after surgery. Malignant tumors should be considered in any patient with chest wall tumors. The thoracoscopic approach could be an optimal treatment for chest wall tumor.

## INTRODUCTION

Primary leiomyosarcomas arising from the chest wall account for ~1–4% of soft tissue sarcomas of the chest wall [1, 2]. We herein describe a unique case of thoracic leiomyosarcoma, which resembled schwannoma on diagnostic imaging. We report the importance of a frozen section examination for its diagnosis and recommend a video-assisted thoracoscopic approach for ensuring an adequate resection margin.

## CASE REPORT

**Figure 1:**
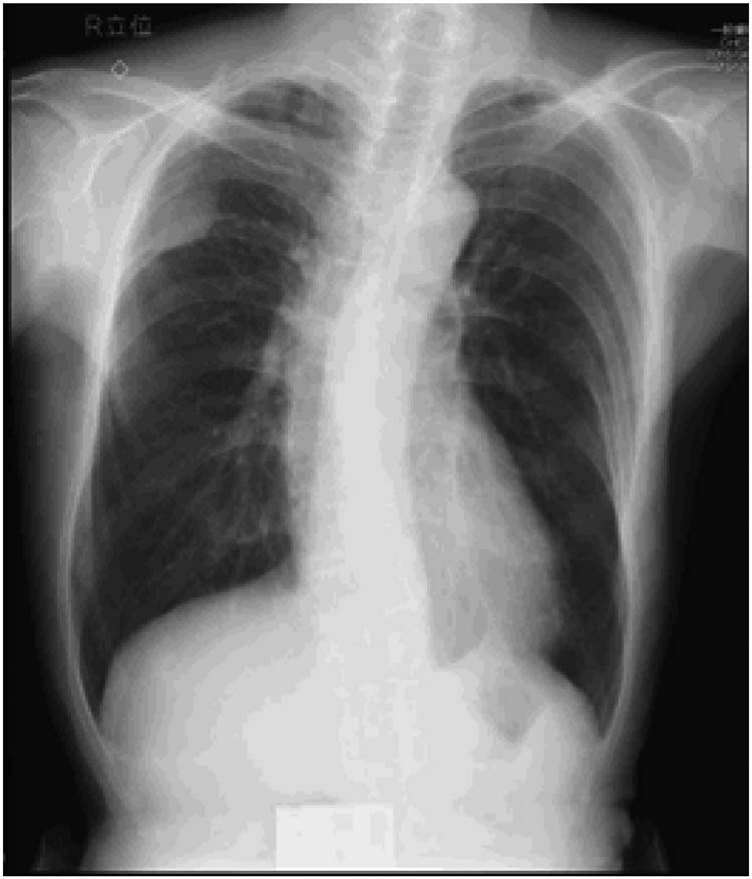
Preoperative chest X-ray: tumor shadow in the right upper lung field.

**Figure 2:**
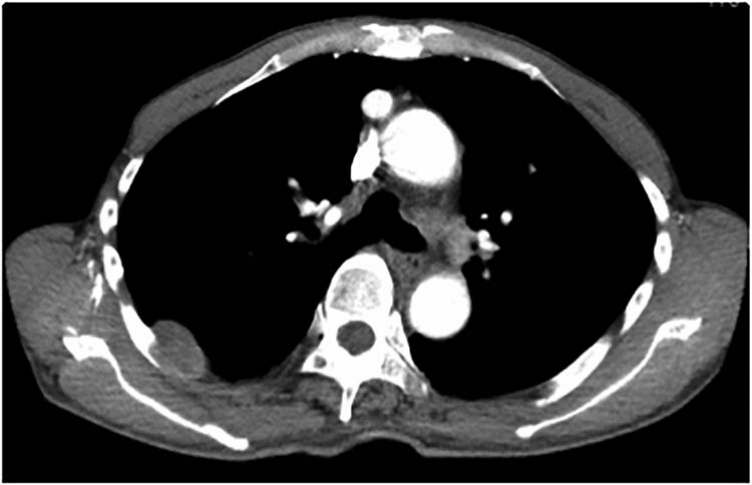
Contrast-enhanced chest CT showing a smooth surface of 3 cm.

**Figure 3:**
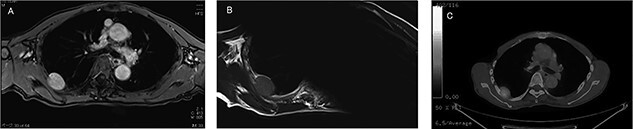
Preoperative MRI and PET-CT: (**A**) contrast-enhanced T1WI: hyper-intense; (**B**) T2WI: iso-intense; (**C**) PET-CT: SUVmax 3.4–4.1.

**Figure 4:**
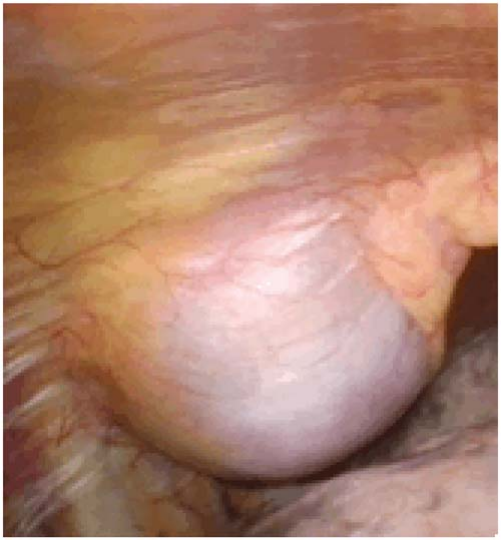
A smooth surface originating from the sixth intracostal space.

**Figure 5:**
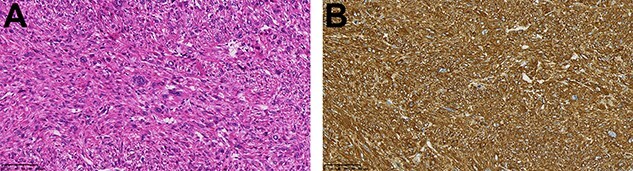
(**A**) The tumor was composed of highly atypical spindle cells (HE staining; magnification: bar = 100 μm); (**B**) α-SMA staining was positive (magnification: bar = 100 μm).

An asymptomatic 79-year-old male who was diagnosed with a chest wall tumor, which had been detected by an X-ray of a health examination, presented to our hospital for surgical intervention. He had no relevant past medical history, including malignant neoplasm or radiation therapy. No mass was palpable. The chest X-ray revealed a tumor shadow of 3 cm in diameter in the right upper lung field (Fig. 1). Chest contrast computed tomography (CT) revealed a well-circumscribed tumor shadow measuring 37 × 27 mm in the posterolateral region of the right sixth intercostal space, the size of which had increased 1.5 times in the previous 3 months (Fig. 2). Thoracic magnetic resonance imaging (MRI) revealed a solid tumor that was enhanced by gadolinium on T1-weighted imaging (T1WI). T2-weighted imaging (T2WI) showed a homogeneous mass with iso-intensity. The tumor was inhomogeneous while demonstrating a maximum standardized uptake value (SUV) of the [18F]-2-deoxy-D-glucose (FDG) uptake under positron emission tomography (PET), with a range of 3.4–4.1 (Fig. 3). There was no significant uptake at other sites, including the mediastinal lymph nodes. No tumor-markers (CEA, proGRP and CYFRA) were detected in a laboratory analysis. Based on these examinations, we suspected a neurogenic tumor, especially schwannoma, and we opted for surgical resection. Considering the fact that ~10% of neurogenic tumors are malignant and the FDG uptake was heterogeneous, we planned to perform an intraoperative frozen section examination. We first performed tumor resection. The operation was performed in the left lateral position under general anesthesia. A 30° viewing angle thoracoscope was set at the middle axillary line of the seventh intercostal space. The tumor was smooth, slightly solid and covered with pleura (Fig. 4). We added a 4-cm incision just above the tumor, while verifying its location through the thoracoscope, and easily removed it from the chest wall. The tumor was diagnosed as sarcoma based on the frozen section examination. Therefore, we added removal of a portion of the sixth and seventh right rib. We excised the chest wall with a 2-cm margin from the lesion, confirming an adequate length through the thoracoscope. Chest wall reconstruction was performed with DUALMESH® (Gore, Flagstaff, AZ). On the cut section, the tumor appeared as solid, smooth-surfaced and encapsulated whitish mass of 3.7 × 2.7 cm in size. Upon microscopic examination, the tumor was composed of fascicles of highly atypical spindle cells mitotic figures exceeding 15 per 10 high-power fields. Immunohistochemistry was positive for αSMA (Fig. 5), desmin and caldesmon and was negative for S-100 protein (data not shown). All margins were negative, and no invasion to the peripheral organs was seen. We concluded that the tumor was leiomyosarcoma of the chest wall. He was discharged on post-operative day 10 without any complications. Since we considered that curative resection had been achieved, no adjuvant therapy was performed. He is still being followed up; however, liver and multiple lung metastases were detected at 5 years after surgery.

## DISCUSSION

Leiomyosarcoma is a malignant neoplasm showing pure smooth muscle differentiation that accounts for 5–10% of all soft tissue sarcomas. It may occur at any anatomical location, but chest wall leiomyosarcoma is rather unusual [3, 4]. Thoracic leiomyosarcomas can result in nonspecific symptoms, such as coughing, chest pain and shortness of breath, or can be asymptomatic [5], as was observed in the present case.

On CT, thoracic leiomyosarcomas are usually characterized by huge soft tissue masses with heterogeneous enhancement because of bleeding, necrosis and other heterogeneous textures. Larger tumors can also push against the surrounding organs and can occasionally be invasive [6)]. Leiomyosarcomas show enhancement on MRI, while they also show a high FDG uptake on PET-CT [7]. Alternatively, schwannoma shows a smooth tumor with homogeneous enhancement on CT. Schwannomas may be heterogeneous due to necrosis or hemorrhage within the mass. On MRI, the tumor is iso- to hypo-intense, while it is also enhanced with contrast media on T1WI and is iso- to hyper-intense on T2WI [8]. The administration of gadolinium causes homogeneous enhancement [9]. In this case, homogeneous enhancement with gadolinium and a clear border were similar to the characteristics of schwannoma. The heterogeneous FDG uptake on PET-CT and rapid growth of tumor were the only findings that raised the suspicion of malignancy.

The optimal treatment for thoracic leiomyosarcoma has not been defined. However, because of its resistance to chemotherapy and radiotherapy, surgery remains the first-line treatment [10)]. The recommended excision range will be similar to that of other sarcomas. Kawaguchi *et al*. reported that a 2-cm margin is acceptable for bone and soft tissue sarcomas [11], while King *et al*. advocated that a 4-cm resection margin leads to a higher 5-year survival rate in thoracic sarcoma [12]. The latest report written by Mesko *et al*. recommended a 2-cm margin for low-grade tumors and a 4-cm margin with R0 resection for high-grade tumors [13]. However, the resection margin depends on the type of tumor and the anatomical location: high-grade histology is associated with a higher rate of recurrence, and complete resection may be difficult when the tumor is in contact with major organs. In this case, despite its high-grade histology, no local recurrence was observed during 5 years of follow-up. Therefore, it is considered sufficient to remove the tumor with 2-cm margin. Setting the resection line while verifying with a thoracoscope is useful for obtaining an appropriate margin.

In summary, the possibility of a malignant chest wall tumor should be considered in any patient with a thoracic mass. We advocate a video-assisted thoracoscopic approach as an optimal approach, which may achieve an adequate resection margin. However, it may take many more years to elucidate the optimal resection margin.

## CONFLICT OF INTEREST STATEMENT

None declared.

## FUNDING

None.
